# SCMPSP: Prediction and characterization of photosynthetic proteins based on a scoring card method

**DOI:** 10.1186/1471-2105-16-S1-S8

**Published:** 2015-01-21

**Authors:** Tamara Vasylenko, Yi-Fan Liou, Hong-An Chen, Phasit Charoenkwan, Hui-Ling Huang, Shinn-Ying Ho

**Affiliations:** 1 Institute of Bioinformatics and Systems Biology, National Chiao Tung University, Hsinchu, Taiwan; 2 Department of Biological Science and Technology, National Chiao Tung University, Hsinchu, Taiwan

## Abstract

**Background:**

Photosynthetic proteins (PSPs) greatly differ in their structure and function as they are involved in numerous subprocesses that take place inside an organelle called a chloroplast. Few studies predict PSPs from sequences due to their high variety of sequences and structues. This work aims to predict and characterize PSPs by establishing the datasets of PSP and non-PSP sequences and developing prediction methods.

**Results:**

A novel bioinformatics method of predicting and characterizing PSPs based on scoring card method (SCMPSP) was used. First, a dataset consisting of 649 PSPs was established by using a Gene Ontology term GO:0015979 and 649 non-PSPs from the SwissProt database with sequence identity <= 25%.- Several prediction methods are presented based on support vector machine (SVM), decision tree J48, Bayes, BLAST, and SCM. The SVM method using dipeptide features-performed well and yielded - a test accuracy of 72.31%. The SCMPSP method uses the estimated propensity scores of 400 dipeptides - as PSPs and has a test accuracy of 71.54%, which is comparable to that of the SVM method. The derived propensity scores of 20 amino acids were further used to identify informative physicochemical properties for characterizing PSPs. The analytical results reveal the following four characteristics of PSPs: 1) PSPs favour hydrophobic side chain amino acids; 2) PSPs are composed of the amino acids prone to form helices in membrane environments; 3) PSPs have low interaction with water; and 4) PSPs prefer to be composed of the amino acids of electron-reactive side chains.

**Conclusions:**

The SCMPSP method not only estimates the propensity of a sequence to be PSPs, it also discovers characteristics that further improve understanding of PSPs. The SCMPSP source code and the datasets used in this study are available at http://iclab.life.nctu.edu.tw/SCMPSP/.

## Background

The photosynthetic conversion of sunlight energy into chemical energy is among the most important biochemical processes on earth. Photosynthetic proteins (PSPs) from plants, algae and photosynthetic bacteria greatly differ in their structure and function as they are involved in many subprocesses, including solar energy harvesting, diffusive transport, energy conversion, electron and ion transport reactions from water to NADP+, ATP generation, and a series of enzymatic reactions in the stroma of the chloroplast [1]. The PSPs are localized in special organelles, called chloroplasts, which have many inner compartments. Although most PSPs are embedded in thylakoid membranes, others are found in the thylakoid lumenal space and in the soluble stroma of chloroplasts. The stromal compartment mostly contains the components of the Calvin cycle, which are needed for fixation of carbon dioxide. The thylakoid membrane contains four protein complexes: photosystem (PS) I, PSII, cytochrome (Cyt) b_6_f, and adenosine triphosphate (ATP) synthase, which carry out the light reactions of photosynthesis [2,3]. The *Arabidopsis thaliana *(*A. thaliana*) genome sequencing project and subsequent proteomics studies have revealed that the thylakoid membrane and thylakoid lumen still contain a number of proteins with unknown functions [4,5]. These proteins may have roles as yet unknown subunits of the photosynthetic complexes and may also be auxiliary proteins guiding the biogenesis, maintenance, and regulated breakdown of the photosynthetic complexes.

Considerable effort is needed to identify novel PSPs using laboratory techniques. Several recent studies have comprehensively analyzed the pea, spinach and *A. thaliana *chloroplast proteome using various fractionation and mass spectrometry methods. Kieselbach *et al*. [3] isolated and characterized the luminal fraction of spinach thylakoids by thylakoid membrane removal, Yeda press fragmentation and centrifugation. Their studies contributed to the discovery of the extrinsic proteins PsbO, PsbP, and PsbQ that are thought to stabilize PSII. In Kleffmann *et al*. [4], tandem mass spectrometry shotgun proteomics was used to develop a comprehensive map of all metabolic and regulatory pathways in *A. thaliana *chloroplasts, which enabled identification of 687 PSPs. Schubert *et al*. [5] studied the chloroplast lumen of *A. thaliana *and used two-dimensional SDS-PAGE, mass spectrometry, and microsequencing techniques for protein separation. Peltier *et al*. [6] identified thylakoid proteome from pea and *A. thaliana *by using gel electrophoresis, mass spectrometry and Edman degradation sequencing. They also presented the results of a stromal proteome analysis of *A. thaliana *in an attempt to quantify proteins of the Calvin cycle [7].

Because of the complexity of chloroplasts and the wide taxonomic distribution among photosynthetic organisms, using only experimental techniques is prohibitively time-consuming and labour-intensive. Therefore, bioinformatics methods have become powerful tools for photosynthetic research. Ishikava *et al*. [8] performed a pilot study that combined bioinformatic and experimental approaches to identify nuclear-encoded chloroplast proteins of endosymbiontic origin. Most proteins in chloroplasts are encoded by the nucleus and require N-terminal presequences (cTPs) to be imported into the organelle [9]. Nakai *et al*. were the first to report protein cTPs in eukaryotic cells [10]. Emanuelsson *et al*. [11,12] proposed neural network-based localization predictors for discriminating cTPs (ChloroP) and for assigning a cleavage site prediction capability (TargetP) to chloroplast, mitochondrion, ER/golgi/secreted, and other localizations. However, not all plastid proteins can be predicted by the localization predictors because several known plastid proteins apparently have no obvious cTPs and because outer envelope proteins of chloroplasts do not have a cleavable cTP [13]. Recent studies have proposed the use of a classifier based on support vector machine (SVM) to identify the four plastid types, including chloroplasts, by utilizing sequence features such as amino acid composition, dipeptide composition, the physicochemical properties of amino acids, etc [14]. However, photosynthetic bacteria have no chloroplasts with photosynthetic proteins directly embedded into plasma membrane. At present, Ashkenasi *et al*. [15] proposed the unique method off identifying PSPs by homology match (BLAST, PSI-BLAST, HSSP, and Pfam). They concluded that, since the false positive rate based on overall sequence similarity is rather high (~70%), short motifs-based approaches can reveal functional similarities more accurately. Therefore, an effective predictor for discriminating between PSPs and non-PSPs from sequences is needed to discover new PSPs for industrial photosynthesis.

Considering the large number of subprocesses in which PSPs are engaged, it is clear that PSPs have a wide range of numerous subprocesses in which they participate, PSPs clearly have widely varying applications. The PSII complex from plants, algae, and cyanobacteria, bacterial reaction centers, and bacteriorhodopsin from halobacteria have the potential to provide the core for numerous innovative devices [16]. The PSII-based biosensors are being developed to replace more complex laboratory analyses used to detect photosynthetic herbicides. Engineered bacterial reaction [centers, as well as OR centers as well as OR centers and the] the isolated components of the PSI are used in photovoltaic cells to promote the conversion of visible light energy into electrical or chemical energy [16]. Another potential application of industrial photosynthesis is producing biodiesel fuel from engineered cyanobacterial organisms [17]. This work had three objectives: 1) developing an effective prediction method for identifying novel PSPs, 2) estimating propensity scores of dipeptides and amino acids to be PSPs for mutagenesis studies, and 3) characterizing PSPs that have potential applications.

Since no dataset of PSPs and non-PSPs is currently available for developing bioinformatics methods that use machine learning, this work first establishes a dataset (PSPGO) consisting of 649 PSPs extracted by using a Gene Ontology term GO:0015979 and 649 non-PSPs from the SwissProt database with sequence identity <= 25%. The proposed SCMPSP prediction method uses the estimated propensity scores of 400 dipeptides as PSPs based on a scoring card method (SCM) [18,19]. The derived propensity scores of 20 amino acids for the 400 dipeptides are then used to discover informative physicochemical properties for characterizing PSPs. To investigate potential prediction methods, several typical prediction methods based on SVM, decision tree J48, and Bayes classifiers with some commonly-used sequence features are also implemented. For comparisons with existing prediction methods, the BLAST method is also implemented [15]. Comparisons of the mean prediction accuracies of all presented prediction methods suggest that the proposed PSPGO dataset provides a higher prediction accuracy compared to the datasetcontaining sequences not reviewed in UniProtKB [15].

To characterize the PSPs, the propensity scores of 20 amino acids were correlated with the physicochemical properties of amino acids in the AAIndex database [20]. Physicochemical properties with high correlation coefficients (*R *values) can be used to study PSPs. However, the reported properties of amino acids for specific protein functions can also be used. The findings of PSP characteristics in this work are as follows: 1) PSPs favour hydrophobic side chain amino acids; 2) PSPs are composed of the amino acids prone to form helices in membrane environments; 3) PSPs have low interaction with water; and 4) PSPs prefer to be composed of the amino acids of electron-reactive side chains.

## Materials and Methods

**Figure 1:**
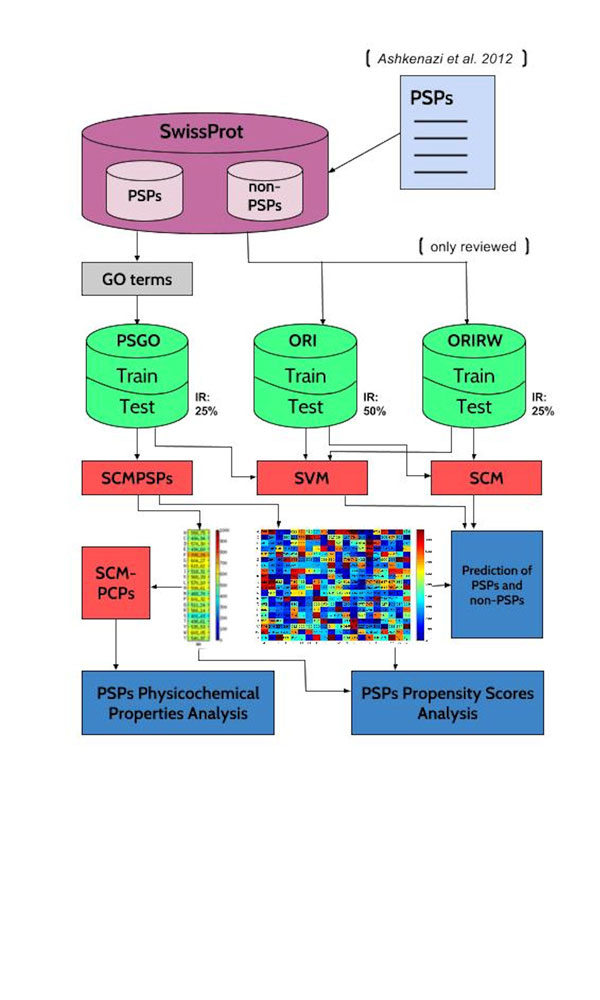
**The flowchart of the system design**.

The proposed SCMPSP method based on a scoring card method (SCM) estimates the propensity scores of 400 dipeptides and 20 amino acids for prediction and characterization of PSPs from their sequences. Figure 1 is a flowchart of the system, including datasets, method, and analysis.

### Datasets

Table 1 shows the three datasets used in this work: PSPGO, ORI and ORIRW. The PSPGO dataset consisting of PSPs and non-PSPs was newly established. Ashkenazi *et al*. [15] provided a dataset of PS proteins without non-PSP sequences collected from the SwissProt. We established both ORI and ORIRW datasets according to this dataset [15].

**Table 1:** Summary of the three datasets consisting of training and test data.

Dataset	Sequence identity (%)	Total	PSP	Non-PSP
PSPGO-TRN	25	1038	519	519
PSPGO-TST	25	260	130	130
ORI-TRN	50	1980	990	990
ORI-TST	50	492	246	246
ORIRW-TRN	25	1000	500	500
ORIRW-TST	25	466	233	233

#### ORI and ORIRW

Ashkenazi *et al*. [15] established the PSP dataset to assess the false positive rate of commonly used homology-based function prediction methods. We adopted this dataset as positive sets of ORI and ORIRW datasets to ensure a reliable performance comparison. However, almost one third of the sequences from the previously reported dataset [15] were marked as 'unreviewed' in the SwissProt. Therefore, the selected PSPs of the ORI dataset included all available PS proteins provided by the previous study [15] (excluding obsolete entries). While the positive part of the ORIRW dataset contained only 'reviewed' PSPs. Putative non-PSPs for both ORI and ORIRW datasets were extracted from the SwissProt and chosen to be all the proteins, originating from the same organisms as those of the PSPs, excluding the positive sequences. The ORI dataset has 1236 positives and 1236 negatives selected randomly from 10692 putative non-PSPs with 50% sequence identity. The ORIRW dataset has 733 reviewed positives and 733 negatives selected randomly from 7048 putative non-PSPs with 25% sequence identity. Both ORI and ORIRW were divided into training (ORI-TRN, ORIRW-TRN) and test (ORI-TST, ORIRW-TST) subsets.

#### PSPGO

Considering the absence of several obsolete entries (from 1425 stated in paper, only 1409 found in SwissProt to date), as well as the presence of 'unreviewed' sequences in the dataset, collected from the previous paper [15] (from 1409 entries 452 'unreviewed'), we established a new PSPGO dataset using the GO terms used to collect unique sequences belonging to the dataset of PSPs, owing to a high quality of its functional annotations.

The PSPGO dataset was used to design the SCMPSP method was established by collecting sequences from the SwissProt database using the GO term GO:0015979 (Photosynthesis) and its child terms, which are not child terms of other processes. The Ancestor chart of the Quick GO browser was used to search relationships among the GO terms. Totally, 30 GO terms were used. The sequences, which were not annotated by these GO terms, were considered putative non-PSPs. The sequence identity of any pair of a sequences was reduced to 25% by using USEARCH [21]. Table 1 shows how the 649 positives and 649 randomly chosen negatives were divided into training PSPGO-TRN and test PSPGO-TST subsets.

## Methods

### Typical classification methods

A literature review shows that few effective methods or tools for predicting PSPs from sequences have been proposed. To develop an accurate predictor, other studies have compared the performance of typical classification methods such as those based on SVM, decision tree J48, and Bayes classifiers with a single type of sequence features. The SVM is generally considered an accurate classifier for predicting proteins with a specific function. Generally, the predictive performance of an SVM with effective features is considered the gold standard for evaluating predictors. Radial basis SVM classifiers are implemented using the LIBSVM package [22]. The SVM parameters are evaluated by using a grid search method to maximize 10-fold cross validation (10-CV) accuracy in a training dataset. Some commonly used features such as amino acid composition (AAC), dipeptide composition (DPC), and 531 PCPs in the AAindex are evaluated in the design of predictors.

The J48 and Bayes classifiers are implemented using WEKA [23]. The J48 is a decision tree classifier generated by the C4.5 algorithm developed by Quinlan [24]. The Naïve Bayes classifier is a statistical classifier that can predict the probability of class membership under the assumption of mutually independent features [25]. For comparison with the existing method [15], BLASTP is used to evaluate the performance of sequence alignment method.

### Scoring card method

The SCM is a new method for predicting proteins with a particular function and for gaining insight into the characteristics of proteins based on OR new method for predicting proteins with a particular function and for characterizing proteins according to primary sequences. Huang *et al*. developed the SCM-based methods [18,26]. Unlike complex classification mechanisms such as SVM, which is not easily understood by biologists, using SCM to estimate the propensities of amino acids and dipeptides to provide the function of interest is a simple and easily interpretable method of prediction and analysis. In terms of prediction accuracy, SCM is slightly worse than, or comparable to, SVM when they are used with dipeptide features [18,26]. The advantages of the SCM method are threefold. First, the classification mechanism of SCM adopts a weighted sum of composition and propensity scores of dipeptides to score the protein sequence. Compared with the hyperplane of the SVM, SCM classifies proteins using a threshold value, which is easily understood and manipulated by biologists. Second, the propensity scores of dipeptides and amino acids can be used to identify the PCPs that provide information about a global property of general proteins in a further analysis of characteristics of the proteins. Third, the SCM is a general-purpose method of identifying protein sequences with a particular function. The proposed SCMPSP method is based on the SCM method using the training dataset of PSPGO-TRN. For a clear understanding, the SCM and the SCMPSP algorithm are described below.

The SCM-based predictors are designed in three main steps: 1) preparing both positive and negative sequences in a training dataset as inputs (519 PSPs and 519 non-PSPs in PSPGO-TRN); 2) using a simple statistical method to generate an initial scoring card with 400 propensity scores of dipeptides; 3) obtaining propensity scores of 20 amino acids from those of 400 dipeptides; 4) using a global optimization method to optimize the initial scoring card, and 5) generating a binary SCM classifier with a threshold value as an output of the procedure. Further details of the SCM method and its applications can be found in [18,26]. The SCMPSP is as follows.

Step 1: Prepare a training dataset PSPGO-TRN comprising 519 PSPs and 519 non-PSPs.

Step 2: Generate an initial scoring card consisting of 400 propensity scores of dipeptides, which are obtained by subtracting the dipeptide composition of dipeptides in non-PSPs from those in PSPs. Then, normalize all dipeptide scores into the range [0, 1000].

Step 3: Calculate the propensity score of each amino acid × by averaging 40 propensity scores of dipeptides that contain X.

Step 4: Use an intelligent genetic algorithm (IGA) to optimize the dipeptide scores [27]. Use the fitness function of IGA to maximize both the prediction accuracy in terms of the area under the ROC curve (AUC) and the Pearson correlation coefficient (R value) between the initial and optimized propensity scores of 20 amino acids. To prevent overfitting, perform a 10-CV assessment to calculate the fitness function as follows (*W*_1 _= 0.9 and *W*_2 _= 0.1 in this study).

(1) $$Max\;Fit\left(Scard\right)=W_{1}\times AUC+W_{2}\times R$$

Step 5: Classify a query sequence *P *based on the scoring function *S(P)*, and determine a threshold value that yields the highest training accuracy. The variables *w_i _*and *S_i _*are the content and propensity score of the *i*-th dipeptide, respectively. Classify *P *as an PSP when *S(P) *exceeds the threshold value; otherwise, classify *P *as a non-PSP.

(2) $$S\left(P\right)=\overset{400}{\underset{i=1}{\sum }}w_{i}S_{i}.$$

### Informative physiochemical properties

Physicochemical properties (PCPs) of amino acids have been shown to have meaningful features that can be used for predicting and analysing the functions of proteins in primary sequences [19]. By using SCMP-PCP [19] to identify the most informative of the 531 PCPs rearranged from the AAIndex that currently contains 544 PCPs of amino acids, PCPs can be discovered, and PSPs can be characterized. Each PCP consists of an accession number, a simple description of the index, the reference information, and the numerical values for the properties of 20 amino acids. The propensity scores of 20 amino acids can be derived from the propensity scores of 400 dipeptides.

The SCM-PCP method is performed in two main steps. First, calculate the *R *value of the Pearson correlation coefficients between the propensity scores and the numerical values of 20 amino acids for each of 531 PCPs. The property of interest is a candidate for PSP function analysis when the absolute value of R is larger than 0.5. 2). Second, use the known PSP function to identify the informative properties from existing studies, which are not included in AAIndex. The composition of amino acids in PSPs and non-PSPs can also be used to infer the properties of PSPs.

## Results and discussion

### Propensity Scores of PSPs

**Figure 2:**
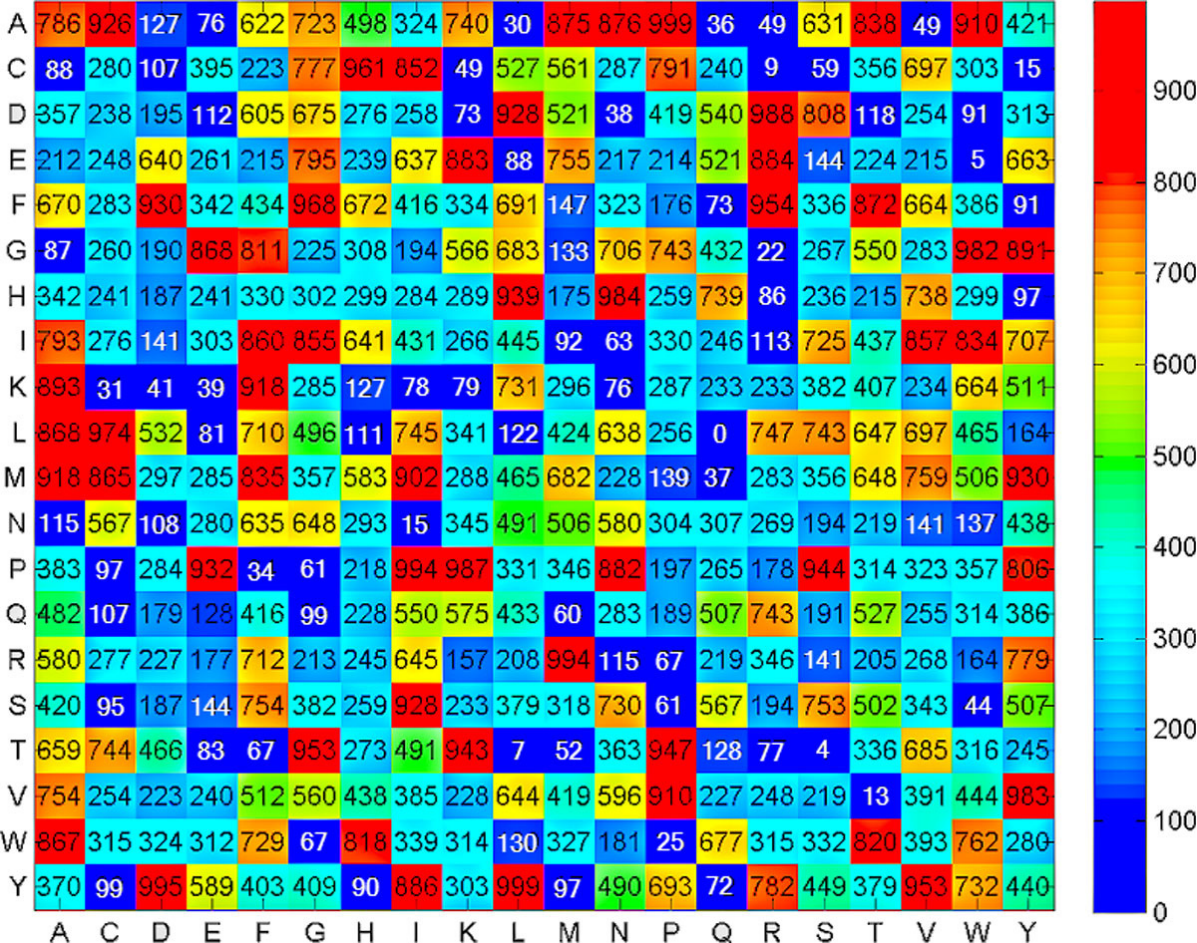
**Heat map of the PSP propensity scores of dipeptides**.

For the proposed SCMPSP method, Figure 2 shows how the training dataset PSPGO-TRN consisting of 519 PSPs and 519 non-PSPs was used to obtain the propensity scores for 400 dipeptides to be PSPs. The dipeptides with the three highest scores are AP, YL and YD (999, 999 and 995, respectively). The dipeptides with the three lowest scores are LQ, TS and EW (0, 4 and 5, respectively). Previous membrane protein studies indicate that membrane-spanning regions are mostly β-turn rich [28] and that the X-Pro motif is contained in the β-turn structure. Since AP, which is an X-Pro motif, has an extremely high score, PSPs have more AP dipeptides compared to non-PSPs, and PSPs may have more β-turn structures compared to non-PSPs.

The PE dipeptide has a rather high score of 932. In Mori *et al*. [29], multiple-sequence alignment of PSPs from plants and bacteria showed that the PE motif is strictly conserved in the transmembrane region of proteins, which is translocated into the thylakoid membrane via the ΔpH-dependent pathway. The PE motif with Glu included in the α-helices of a transmembrane region apparently stabilizes the protein structures by forming a stable interaction with Lys, His or Gln. Even in proteins such as Tha4 and TatA/E, which have different functions, this protein is still conserved. The GP motif, which has a fairly high propensity score of 743, was also completely conserved between the membrane-spanning helix and amphipathic helix of ΔpH-dependent protein precursors [29]. The twin arginine motif RR was a distinguishing feature in protein precursors translocated into the thylakoid membranes via the ΔpH-pathway. The three dipeptides PE, GP, and RR have important roles in the ΔpH-pathway.

**Figure 3:**
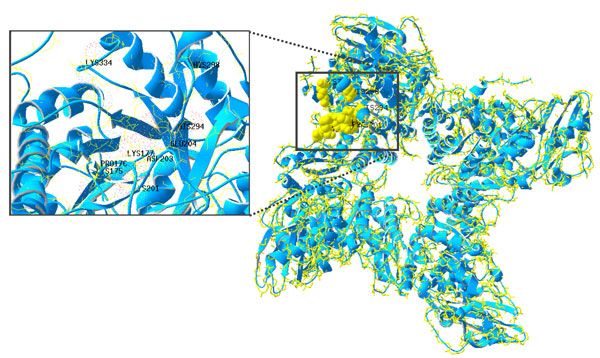
**Structures of *Pisum sativum *Rubisco**. PDB entry 4HHH. Molecular structures of the residues of active center are highlighted in solid yellow. Detailed residue positions are shown as spacefill. Figures were made by Swiss-PdbViewer 4.1.0.

**Figure 4:**
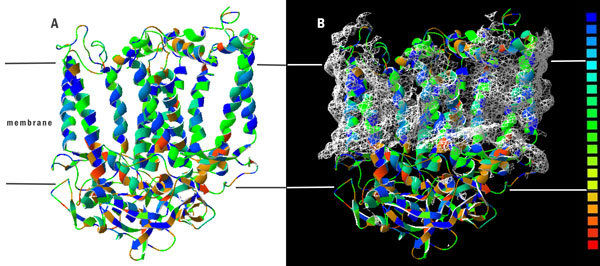
**Structures of *Rhodobacter Sphaeroides *Reaction Center**. PDB entry 2WX5. (A) Distributions of side chain hydrophobicities; (B) Hydrophobic patches highlighted. Side chains colored according to hydrophobicity palette with blue at the hydrophobicity extreme, green in intermadiate, red for hydrophilic side chains. Figures were made by Swiss-PdbViewer 4.1.0.

Table 2 presents the 20 amino acid compositions of PSPs and non-PSPs calculated from 133,744 and 210,361 residues, respectively. The high correlation coefficient of *R *= 0.96 between the propensity scores of amino acids and composition difference of amino acids between PSPs and non-PSPs indicates that propensity scores of amino acids are effective for discriminating between PSPs and non-PSPs. The five top-ranked amino acids are Ala, Phe, Tyr, Ile and Leu whereas the residues with the lowest propensity scores are Lys, Asp, Arg, Glu, and Gln. Most of the top-ranked amino acids are are hydrophobic amino acids. One exception is Ala, which has a scale close to the hydrophobic/hydrophilic threshold in many well-known hydrophobicity scales [30]. We postulate that PSPs are composed of hydrophobic amino acids for two main reasons. First, in the main photosynthesis mechanism, membrane and transmembrane proteins have important roles in energy generation reactions such as electron transfer and as a driving force in the pH gradient. In transmembrane proteins, photosynthesis must occur in an extremely hydrophobic environment. The PSPs consist of hydrophobic amino acids that reduce the protein folding free energy that affect the stability of protein structures. As expected, hydrophilic amino acids such as Gln, Glu, Arg and Asp have extremely low propensity scores, which suggest that PSPs do not favour the composition of hydrophilic amino acids. As Figure 3 shows, the four main protein complexes involved in the light reactions of photosynthesis are embedded in the thylakoid membranes of chloroplasts [31,32]. Two photosystems are needed move electrons from water to Nicotinamide adenine dinucleotide phosphate (NADPH) [33]. Photosystem II (PSII) catalyses the light-induced transfer of electrons from water to plastoquinone and releases oxygen [16]. The hydrophobic core of 20 proteins in this protein complex is surrounded by a specific light-harvesting system (LHCII) [31]. The D1 and D2 core proteins are components of the reaction center, and each possesses five hydrophobic transmembrane helices [31]. Photosystem I (PSI) operates in association with PSII to generate NADPH. The backbone of PSI is a heterodimer consisting of two subunits called PSI-A and PSI-B [32]. These two subunits contain 11 hydrophobic domains, which form eight transmembrane helices plus two large surface helices lying parallel to the membrane plane. This central heterodimer binds 12 small membrane-embedded proteins (core complex) and is surrounded by the light-harvesting complex (LHCI) [33]. Additionally, Rees *et al*. [34] conducted a study of hydrophobic organization of the 11 transmembrane a helices of the photosynthetic reaction center from *Rhodobacter sphaeroides *and showed that membrane-exposed residues are more hydrophobic than buried interior residues (Figure 4).

**Table 2:** The propensity scores and composition (%) of amino acids.

Amino acid	PS protein Score (Rank)	Composition of PS: A(%)	Composition of Non-PS: B(%)	Composition difference: A-B(%)
A-Ala	522.90 (1)	9.97	8.03	1.94
F-Phe	516.70 (2)	5.12	3.68	1.43
Y-Tyr	498.90 (3)	3.34	2.68	0.66
I-Ile	495.80 (4)	6.36	5.41	0.95
L-Leu	484.90 (5)	11.28	10.20	1.08
G-Gly	482.70 (6)	7.70	6.95	0.75
V-Val	448.60 (7)	7.45	6.73	0.72
M-Met	447.70 (8)	2.77	2.26	0.51
P-Pro	429.30 (9)	4.72	5.09	-0.36
W-Trp	417.40 (10)	1.29	1.14	0.15
T-Thr	413.60 (11)	5.07	5.21	-0.14
S-Ser	383.60 (12)	6.79	7.40	-0.61
N-Asn	376.10 (13)	3.40	3.85	-0.45
H-His	373.30 (14)	1.75	2.33	-0.58
C-Cys	371.10 (15)	1.06	1.06	-0.69
K-Lys	370.70 (16)	4.58	5.51	-0.93
D-Asp	358.80 (17)	4.35	5.24	-0.89
R-Arg	356.70 (18)	4.80	5.78	-0.98
E-Glu	350.90 (19)	5.33	6.64	-1.31
Q-Gln	313.10 (20)	2.90	4.12	-1.23

R	1.000	0.53	0.22	0.96

The total numbers of amino acids for PSPs and non-PSPs in PSPGO-TRN are 133,744 and 210,361, respectively.

Second, PSPs must build hydrophobic environments for binding with coenzymes and cofactors such as heme and chlorophyll. For example, most electron transfer reactions during photosynthesis result from protein binding with chlorophyll, which is a porphyrin derivative. The PSPs with the chlorophyll as the cofactor form the transmembrane α-helical structures, which favour Ala as the reaction center (review see [34]). In this reaction center, the top-ranked residues in terms of composition are Leu (15%), Ala (14%), Phe (12%) and Ile (10%), all of which are hydrophobic amino acids.

Nagata *et al*. [35] first reported the LH-α polypeptide, a light harvest [4] peptide, which can assemble the LH complex OR a light harvest [4] peptide that can assemble the LH complex adjacent to the photosynthesis reaction center. The LH peptides have three main segments: N-terminal, hydrophobic core, and C-terminal. Ochiai *et al*. [36] attempted to use various LH1-β peptides and their derivatives, which have the same hydrophobic core with variation in N- and C-terminal, to form self-assembled monolayers (SAMs) of organic molecules. Their experimental results suggest that the hydrophobic core of the LH polypeptides is invariable while the N- and C-terminal is variable. The result shows the efficient energy transfer and electron transfer reactions between individual pigments on the electrode. Table 3 shows that, in the hydrophobic cores of the LH polypeptides that self-assemble, the hydrophobic cofactor interacts with the hydrophobic cofactor.

**Table 3:** The hydrophobic core of the light harvesting polypeptide.

Name	sequence	reference	Propensity score
LH1-b	VYMSGLWLFSAVAIVAHLAVYIW	[54]	554.77
LH-a	ALVGLATFLFVLALLIHFILLST	[54]	518.91
Cut-a	ALVGLATFLFVLALLIHFILLST	[54]	518.91
Type1	ALVGLATFLFVLALLIHFILLST	[54]	518.91
LH-b	IFTSSILVFFGVAAFAHLLVWIW	[55]	604.77

The mean score of 400 dipeptide propensity is 420.27 and the threshold of the SCMPSP classifier is 441.29.

The lowest score for these five hydrophobic cores is 518.91, which is much higher than the threshold 441.29 of the PSP classifier. In this LH polypeptide, Leu has the highest average composition (20.23%) followed by Ala (14.50%). The hydrophobic cores revealed no extreme polar residues such as Asp, Asn, Glu and Gln.

### Performance comparisons of PSP predictors

Three datasets were used to design various PSP classifiers and to compare prediction performance in all classification methods with various feature types. The proposed SCMPSP method was compared with SVM, decision tree J48, and Bayes classifiers. For each classifier, we evaluated three kinds of features: 1) amino acid composition (AAC), 2) dipeptide composition (DPC), and 3) 531 PCPs in AAindex.

The first comparison was with methods that use BLAST [15], a commonly-used strategy for classifying proteins based on sequence similarity. The BLAST-based method BLSTP estimates the performance of a sequence alignment method. This method uses similar regions or segments of sequences, which are referred as "hot spots" on the sequence, and extends the other regions of the query protein. The training datasets are used to build sequence database for BLSTP. The test dataset is used as query sequences to align against the database. The E-value ranges from 0.1 to 0.00001. The sequences with the highest score are used to classify the query sequence. Table 4 shows the three training datasets PSPGO-TRN, ORI-TRN, and ORIRW-TRN corresponding to the three test datasets PSPGO-TST, ORI-TST, and ORIRW-TST. The BLSTP method obtains the best results when the e-value is 0.01. For PSPGO-TST, ORI-TST, and ORIRW-TST, the BLSTP has accuracies of 58.46%, 40.85%, and 32.40%, respectively, which are much smaller than those of SCMPSP, SVM, decision tree J48, and Bayes classifiers.

**Table 4:** Performance of established datasets as compared for various E-value cut-offs by BLSTP

e-value	PSPGO-TST	ORI-TST	ORIRW-TST
0.1	58.46%	40.85%	32.40%
0.01	57.31%	38.21%	30.26%
0.001	56.15%	36.99%	28.76%
0.0001	53.46%	35.98%	27.47%
0.00001	51.92%	34.15%	26.39%

Table 5 compares the prediction accuracies of SCMPSP and other methods with the three features. The comparison results show that PSPGO-TST has the highest test accuracy in all three datasets. Themean test accuracies of PSPGO-TST, ORI-TST, and ORIRW-TST are 68.85%, 55.23%, and 57.54%, respectively. The SVM-based method performed well, and the SVM-based, decision tree J48-based, and Bayes-based methods with three feature types had mean test accuracies of 64.93%, 57.84%, 56.04%, respectively. The mean test accuracy is higher with the dipeptide features (60.20%) than with the amino acids features (60.20%). The comparisons reveal that SVM-based methods, dipeptide composition features, and PSPGO-based datasets can be used to develop effective methods of predicting PSPs.

**Table 5:** Comparison of the prediction accuracies (%) of PSP predictors.

Classifier	PSGO-TRN	PSGO-TST	ORI-TRN	ORI-TST	ORIRW-TRN	ORIRW-TST	Mean
SCMPSP	83.82	71.54	77.78	62.60	81.8	64.38	66.17
SVM-AAC	85.45	78.85	71.36	50.61	71.20	57.30	62.25
SVM-DPC	83.33	72.31	68.94	69.11	67.30	61.37	67.60
SVM-AAindex	79.19	75.38	71.40	71.03	71.36	71.14	72.52
J48-AAC	68.50	73.84	64.24	63.41	68.21	49.79	62.35
J48-DPC	63.20	61.92	55.25	51.22	55.70	59.44	57.53
J48-AAindex	65.03	70.38	62.50	62.02	61.46	68.09	66.83
Bayers-AAC	67.92	69.20	64.65	63.00	65.00	65.02	65.74
Bayers-DPC	65.41	67.31	58.28	57.32	58.80	60.73	61.79
Bayes-AAindex	66.09	64.62	62.70	60.30	62.73	57.11	60.68

Mean	72.79	70.54	65.71	61.06	66.37	61.44	

The experimental results can be briefly summarized as follows. The performance of SCMPSP, SVM-based, J48-based, and Bayes-based methods to predict PSPs outperform BLSTP. The PSPGO datasets are more suitable to develop methods of predicting PSPs than the ORI and ORIRW datasets. For predicting PSPs, SVM-based methods are more effective than J48- and Bayes-based methods. Bayes-based methods do not perform well in predicting PSPs. The performance of the SCMPSP method is comparable to that of the SVM-DPC method, which outperforms J48-DPC and Bayes-DPC in the PSPGO-TRN and PSPGO-TST datasets.

### Performance evaluation of SCMPSP

Table 6 presents the results of ten independent runs of SCMPSP on the PSPGO dataset consisting of PSPGO-TRN and PSPGO-TST. Experiment 7 has the highest training accuracy (83.82%), and the corresponding test accuracy, sensitivity, specificity, AUC, and threshold are 71.54%, 0.7154, 0.7154, 0.9, and 441.29, respectively. The optimization approach improves the training accuracy 15.03% from 68.79% to 83.82%.

**Table 6:** 10 independent runs of the SCMPSP on PSPGO-TRN.

#	Fitness Score	Train Accuracy (%)	Sensitivity	Specificity	Threshold
1	0.9016	82.5626	0.7154	0.6615	459.0526
2	0.9097	82.0809	0.7231	0.6538	454.5208
3	0.9105	83.8150	0.6615	0.6308	460.6491
4	0.9022	82.9480	0.6692	0.7231	429.4792
5	0.9136	83.6224	0.6462	0.7385	465.0526
6	0.9051	82.6590	0.6700	0.5923	456.3793
7	0.9114	83.8150	0.7154	0.7154	441.2917
8	0.9046	82.4663	0.6615	0.6700	456.5833
9	0.9027	81.6956	0.7231	0.6846	441.4901
10	0.9088	82.5626	0.7308	0.6000	448.3220

Mean	0.9070	82.8227	0.6916	0.6670	451.2821
STDV	0.0043	0.7253	0.0325	0.0500	10.9653

The SCMPSP method achieves a test accuracy of 71.54%, an MCC of 0.43, a sensitivity of 0.72, and a specificity of 0.72 in PSPGO-TST. The SVM-DPC method using SVM with DPC features achieves a test accuracy of 72.31%, an MCC of 0.45, a sensitivity of 0.75, and a specificity of 0.70. These experimental results indicate that SCMPSP is comparable to SVM-DPC in terms of predicting PSPs.

### Propensity analysis using informative PCPs

Table 7 shows the tree physicochemical properties (PCPs) selected by SCM-PCPs from the AAindex database. The correlation between these PCPs and the propensity scores derived from SCMPSP is evaluated with the Pearson correlation coefficient (the R value). The three PCPs are BLAS910101 (R = 0.7955), PUNT030101 (R = -0.7948) and WOLR810101 (R = 0.7597). The analysis results for the three PCPs for PSPs are discussed below.

**Table 7:** The amino acids scores derived from SCMPSP and physicochemical properties selected by SCM-PCPs.

Amino acid	PS protein Score (Rank)	^1^BLAS910101 Score (Rank)	^2^WOLR810101 Score (Rank)	^3^PUNT030101 Score (Rank)
A-Ala	522.9 (1)	0.62 (10)	1.95 (5)	-0.17 (15)
F-Phe	516.7 (2)	1.00 (1)	-0.76 (6)	-0.41 (20)
Y-Tyr	498.9 (3)	0.88 (4)	-6.11 (13)	-0.09 (13)
I-Ile	495.8 (4)	0.94 (2)	2.15 (3)	-0.28 (18)
L-Leu	484.9 (5)	0.94 (3)	2.28 (2)	-0.28 (19)
G-Gly	482.7 (6)	0.50 (11)	2.39 (1)	0.01 (10)
V-Val	448.6 (7)	0.83 (6)	1.99 (4)	-0.17 (16)
M-Met	447.7 (8)	0.74 (7)	-1.48 (8)	-0.26 (17)
P-Pro	429.3 (9)	0.71 (8)	-3.68 (9)	0.13 (7)
W-Trp	417.4 (10)	0.88 (5)	-5.88 (12)	-0.15 (14)
T-Thr	413.6 (11)	0.45 (12)	-4.88 (10)	0.02 (9)
S-Ser	383.6 (12)	0.36 (13)	-5.06 (11)	0.05 (8)
N-Asn	376.1 (13)	0.24 (16)	-9.68 (16)	0.18 (5)
H-His	373.3 (14)	0.17 (17)	-10.27 (18)	-0.02 (11)
C-Cys	371.1 (15)	0.68 (9)	-1.24 (7)	-0.06 (12)
K-Lys	370.7 (16)	0.28 (14)	-9.52 (15)	0.32 (3)
D-Asp	358.8 (17)	0.038 (19)	-10.95 (19)	0.37 (1)
R-Arg	356.7 (18)	0.00 (20)	-19.92 (20)	0.37 (2)
E-Glu	350.9 (19)	0.04 (18)	-10.20 (17)	0.15 (6)
Q-Gln	313.1 (20)	0.25 (15)	-9.38 (14)	0.26 (4)

R	1.000	0.7955	0.76	-0.79

^1^BLAS910101 = Scaled side chain hydrophobicity values (Black-Mould, 1991).

^2^WOLR810101 = Hydration potential (Wolfenden et al., 1981).

^3^PUNT030101 = Knowledge-based membrane-propensity scale from 1D_Helix in MPtopo databases (Punta-Maritan, 2003).

#### A. PSPs favour hydrophobic side chain amino acids

The BLAS910101, which can be described as ''Scaled side chain hydrophobicity values'', had the highest positive correlation (R = 0.7955) [37]. Estimation of hydrophobicity profiles has proven to be a powerful approach to protein sequence analysis. Many scales have been developed to quantify the hydrophobic properties of the standard 20 amino acids. The values of BLAS910101 property were calculated by using a modification of the 'hydrophobic fragmental constant' approach developed by Rekker [6], *i.e.*, only the side chains of the post- or cotranslationally modified amino acyl residues were considered and not the peptide backbone [37].

The high positive correlation indicates that PSPs favour hydrophobic amino acids. Table 7 shows that the top five amino acids are Ala, Phe, Tyr, Ile and Leu. According to the property BLAS910101, four of these residues (Phe, Leu, Ile and Tyr) possessed the highest side chain hydrophobicity values. However, the Ala residue had a rank of 10] [37].

As mentioned in the primary analysis of the propensity scores, the overall hydrophobicity of the PSPs is high. In SCM-PCPs, however, the hydrophobic characteristics of the side chains do not include the backbone hydrophobic characteristics because BLAS910101 does not include the backbone hydrophobicity. Based on these experimental results, we postulate that side chains play an important role in reducing the folding free energy and to construct the hydrophobic environments for cofactor binding.

**Figure 5:**
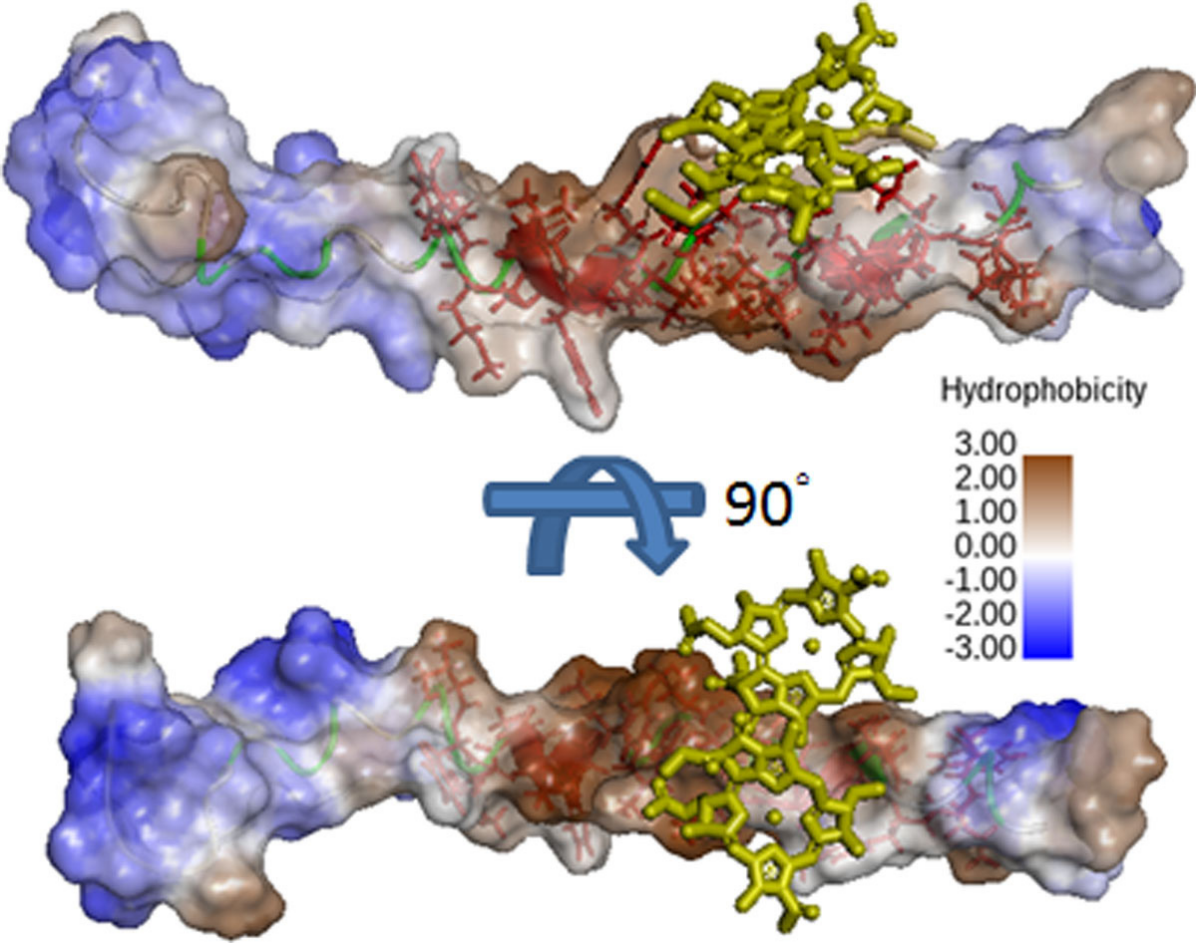
**The structures of light harvesting peptide, LH1, and cofactor, bacteriochlorophyll A**. The peptide structure is extracted from the light harvesting complex containing 64 peptides, 4JC9. The cofactors (yellow sticks) contacting with it are also extracted. Red sticks denote the hydrophobic core residues. The colors of the surface from brown (the most hydrophobic) to blue (the most hydrophilic) indicate the hydrophobicity. The graph is generated from the Discovery Studio 4.0.

The high correlation between the property BLAS910101 and propensity scores of PSPs indicates that the side chain hydrophobicity is important to PSPs. As noted above, the LH polypeptides have a hydrophobic core for porphyrin-derivative binding. For further insight into their structure, PDB structures containing the LH1-β hydrophobic core sequence VYMSGLWLFSAVAIVAHLAVYIW is used in a PDB database search. The 4JC9, an LH complex containing 64 LH peptides and 128 cofactors, is then selected. Of 14 peptides containing the LH1-β hydrophobic core sequences, one is used for further analysis. The selected peptides have a score of 496.90, which is higher than the SCMPSP threshold of 441.29 but lower than the hydrophobic core score of 554.77. Figure 5 shows that the LH1-β hydrophobic core sequence forms helix-like structures. The sequence shows a gradient hydrophobicity that the two tails of this peptide show a hydrophilic character, but the middle of the sequence shows an extremely hydrophobicity. The middle segment uses the side chain to to form the hydrophobic surface, and the cofactors are in contact with a hydrophobic side chain. These sequencing results suggest that the side chain can construct the hydrophobic environment for cofactor binding and can interact with the cofactors.

#### B. PSPs are composed of the amino acids prone to form helices in membrane environments

The PUNT030101 property, described as ''Knowledge-based membrane-propensity scale from 1D_Helix in MPtopo databases'', showed the highest negative correlation (R = -0.7948) [38]. Of the many hydropathy scales currently available for protein analysis, one of the most widely used is the knowledge-based amino acid membrane-propensity scale developed by Punta *et al*. [38], which exhibits strong correlation with several representative hydropathy scales, and approaches different prediction tasks. This scale is derived using a set of transmembrane helices segments from MPtopo databases with the requirement that each component of the set must have an OR database. However, each component of the set must have a free energy lower than that of a typical soluble protein sequence of the same length [38]. Punta *et al*. attempted to use this index to solve two problems: predicting the soluble/membrane proteins and predicting α-helical transmembrane/signal segments. Hence, PUNT030101 has two characteristics: the propensity to form a membrane and the propensity to form an α-helical structure in the membrane.

High correlation with PUNT030101 suggested that PSPs tend to be composed of amino acids with high membrane propensity. Since negative values for PUNT030101 indicate high membrane propensity, a strong inverse correlation between SCMPSP and PUNT030101 scores implies a high membrane propensity of the photosynthetic proteins. Table 7 shows that the amino acids with the 5 highest propensity scores correspond to the bottom- and middle-scored Phe, Leu, Ile, Ala and Tyr residues at ranks 20, 19, 18, 15 and 13, respectively. In contrast, those with the lowest scores were Gln, Glu, Arg, Asp and Lys, with PUNT030101 ranks of 4, 6, 2, 1 and 3, respectively. Notably, only Lys is a hydrophobic amino acid. A high hydrophobicity is a characteristic feature of amino acids belonging to transmembrane regions, which is in agreement with the previously described BLAS910101 property correlation results [38]. Figure 3 shows that chlorophyll-protein complexes, which catalyze the light reactions of the photosynthesis, are known to be embedded in the thylakoid membranes of chloroplasts. The photosynthetic thylakoid membrane encloses a single lumen space and differentiates into cylindrical stacked grana and interconnecting single membrane regions called the stroma lamellae. The four membrane-associated components of the photosynthetic apparatus include PSI and ATPase, which are located in the stroma lamellae of thylakoids, PSII, which resides mainly in the grana membranes, and the cytochrome b6/f complex, which is almost evenly distributed between the two membrane types [2]. In each case, the overall organization and the number of transmembrane regions differ. Remarkably, both PSI and ATPase complexes have bulky stromal-exposed parts whereas the PSII core and cytochrome b6/f complexes protrude from the lumenal side [2]. These results indicate the important role of the membrane in photosynthesis, so some PSPs must have functions in the membrane environment.

The high correlation with PUNT030101 also indicates that PSPs tend to be composed of amino acids that form transmembrane helices. In the photosynthetic reaction centers (RCs), the membrane-spanning helices are the main structures. For example, the RCs from purple bacteria have 11 membrane-spanning helices that can form a hydrophobic binding site for cofactor binding; in contrast, some external helices that are exposed outside the membrane connect to the membrane-span helices.

#### C. PSPs have small interaction with water

The property of WOLR810101, described as "Hydration potential", was selected by SCM-PCPs with R = 0.7597 [39]. The hydration potentials of amino acid side chains represent their free energies of transfer from the vapor phase to dilute water. The structures of macromolecules can be determined by comparing amino acid residues in terms of their strength of solvation by water. Many researchers have attempted to calculate solvation properties of the natural amino acid side chains. By using more sensitive techniques compared to earlier measurements, Wolfenden *et al*. developed a scale of effective free energies of transfer of amino acid side chains from the vapor phase to neutral aqueous solution buffered at pH 7. Since highly hydrophilic amino acid side chains strongly release energy after they are dissolved in water, those lower score amino acids in WOLR810101 indicated these residues favour interaction with water molecules and *vice versa*. Wolfenden *et al*. reported the results for a scale of hydration potential spanning a range of ~22 kcal/mol. The residues with the five lowest scores were Arg, Asp, His, Glu and Asn [39].

Table 7 shows that the residues with the five lowest propensity scores were hydrophilic residues Gln, Glu, Arg, Asp and Lys, with Arg being at rank 18. According to the previously reported scale, aliphatic side chains of Gly, Leu, Ile, Val and Ala can only form weak bonds with water and exhibited positive free energies of transfer from vapor phase to water [39]. Among the SCMPSP top-5 scored residues, Ala, Ile and Leu are found together with aromatic Phe and Tyr residues. The Phe and Tyr were ranked 6 and 13, respectively, by a previously reported scale of amino acid affinities. Wolfenden *et al*. not only determined affinities of amino acid side chains, but also identified a statistically significant relationship between the resulting scale and the inside-outside distributions of amino acids in globular proteins. The outside residues directly interact with water while the inside ones do not. Based on earlier solvent accessibility calculations, they showed that residues with negative free energies of transfer from the vapor phase to water tend to appear on the surface rather than in the interior of globular proteins [9]. Thus, unlike the accessible Arg, Asp, His, Glu and Asn residues, the Gly, Leu, Ile, Val and Ala amino acids tend to be "buried".

**Figure 6:**
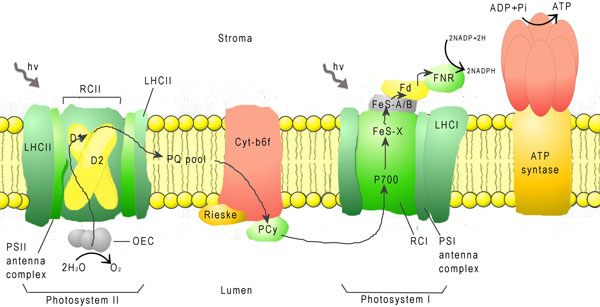
**A schematic representation of the photosynthetic apparatus in the thylacoid membrane**.

The strong positive correlations between reported hydration potentials scale and SCMPSP propensity scores indicated that the amino acid interact with water molecules do not prefer to be the elements of PSPs. This phenomenon leads us to the assumption that the PSPs less interact with water molecules. Figure 6 shows that the weak interaction between PSPs and water molecules results from the photosynthetic process in thylakoid. The PSPs often work with co-enzymes such as Cyt-b6f and the PSII antenna complex and need to work with plastoquinone (PQ) while the PSI antenna complex works with NADP. The movable electrons are generated from water by the PSII antenna complex in PSII. After the electron is transported to PQ and plastocyanin (PCy), PCy carries the electron to PSI and then transfers the electron to NADP. The coenzymes PQ and NADP are hydrophobic molecules, and the interaction with the water molecules would create a polar environment that is unfavorable to such hydrophobic molecules. The composition of the amino acid residues and the low interaction with water keeps the environment of electron transport chain stable and efficient.

#### D. PSPs tend to be composed of amino acids with electron-reactive side chains

Photosynthetic machinery that collects solar energy and converts it to chemical energy is susceptible to oxidative damage resulting from an excess light [40,41]. Strong light causes oxidation and increases production of reactive oxygen species [42]. Many studies of the effects of reactive oxygen species (ROS) on various proteins [43-45] indicate that ROS cause oxidative damage to proteins, which results in biological dysfunctions such as perturbed activity of enzymes, transport proteins and receptors [46], which can oxidatively modify the proteins and enhance the proteolysis process in orgasms. ROS are also generated by the light-driven reactions of electron transfer decrease PSII activity and lead to irreversible oxidation of the D1 protein in photosynthesis systems [47]. To reduce ROS damage, some ROS catalytic enzymes such as glutathione peroxidase and superoxide dismutase have been studied because they apparently have important roles in destroying ROS and the interplay between cyclic, linear and pseudocyclic electron transport pathways is required for the prevention of over-oxidized state and coordination of energy metabolism during photosynthesis [40]. Some studies of mechanisms that overcome the oxidative stress have postulated that the photosynthesis system has a prevention mechanism in which the PSPs can immediately capture the ROS generated from light, and prevent the ROS to attack other PSPs. However, the AAindex database does not have an ROS-related index.

As expected, the extent to which different amino acid side chains are oxidized varies. Davies *et al*. attempted to quantify these differences by using a scale of rate constants for reaction of the hydroxyl radical (HO*), which is among the most reactive and least selective ROS, with free (zwitterionic) amino acids, and small peptides, at pH ca. 7 [48]. Table 8 shows that our comparisons of this scale with the propensity scores obtained from the SCMPSP method obtained slightly positive correlation results (R = 0.3146). Since the oxidant reaction rate constants are measured from the side chain oxidation, the influences of side chain would be considered. The Gly, Pro and Ala eliminate the side chain beyond the β carbon and would not provide additional side chain effects. Further analysis led us to hypothesize that the functional group of side chain would be more important to react with the ROS. Hence, the three amino acids Ala, Pro and Gly, which lack side chain effects, are not capable of reacting with electrons.

**Table 8:** The SCMPSP scores and Rate constants by Davies et al. [48]

Amino acid	PS protein Score (Rank)	**Rate constants by Davies et al. **[48]
A-Ala	522.9 (1)	7.7 × 10^7^
F-Phe	516.7 (2)	6.5 × 10^9^
Y-Tyr	498.9 (3)	1.3 × 10^10^
I-Ile	495.8 (4)	1.8 × 10^9^
L-Leu	484.9 (5)	1.7 × 10^9^
G-Gly	482.7 (6)	1.7 × 10^7^
V-Val	448.6 (7)	7.6 × 10^8^
M-Met	447.7 (8)	8.3 × 10^9^
P-Pro	429.3 (9)	4.8 × 10^8^
W-Trp	417.4 (10)	1.3 × 10^10^
T-Thr	482.7 (11)	5.1 × 10^8^
S-Ser	448.6 (12)	3.2 × 10^8^
N-Asn	447.7 (13)	4.9 × 10^7^
H-His	429.3 (14)	1.3 × 10^10^
C-Cys	417.4 (15)	3.4 × 10^10^
K-Lys	370.7 (16)	3.4 × 10^8^
D-Asp	358.8 (17)	7.5 × 10^7^
R-Arg	356.7 (18)	3.5 × 10^9^
E-Glu	350.9 (19)	2.3 × 10^8^
Q-Gln	313.1 (20)	5.4 × 10^8^

R1^a^ R2^b^	1.00 1.00	0.31 0.50

a. all amino acid residues

b. excluding the amino acids lacking the side chain effect

Therefore, Ala, Pro and Gly were excluded from further correlation analysis. After excluding these three amino acids, the correlation dramatically increases from 0.31 to 0.50. This phenomenon indicates that PSPs tend to be composed of amino acids with high ROS reacting side chains.

In the ROS reaction constant analysis, Ala has the highest propensity score, but its side chain has a weak interaction with ROS. Some researchers have hypothesized that most PSPs function in the membrane environment and are composed of the alpha-helical structures. Arkin and Brunger [49] provided statistical results showing that Ala tends to be composed of transmembrane alpha-helical structures since structures that influence protein functions are more important for PSPs than for capturing ROS. The Trp with a rapidly ROS interacting ability is rank tenth, which indicates that PSPs and non-PSPs would have equal propensity to be composed of Trp. Although the experiments show that Trp has a good ability to react with ROS, Trp is a high energy amino acid that needs most energy to be produced. This explains why PSPs do not use this residue to protect against ROS.

The correlations obtained for the highly oxidizing HO* radical indicate that the PSPs tend to be composed of amino acids that deplete rapidly. The advantage of this amino acid composition is the neutralization of the ROS that cause protein damage and prevent ROS from destroying the photosynthesis system. Studies of the cell cycle also indicate that an overdose of ROS can trigger apoptosis. (review see [50]) Although live organisms have enzymes such as glutathione peroxidase and super oxide dismutase that can catalyze ROS, rapidly neutralizing ROS may be a good strategy for preventing ROS accumulation. In the photosynthesis system, however, generation of the energy and oxidative stress uses the energy saving strategy that Trp, an energy cost amino acid but having better ability to absorb the ROS, is not preferential in PSPs compared to non-PSPs.

The above understanding is needed to engineer photosynthetic organisms with enhanced oxidative stress tolerance [51]. Therefore, plants with enhanced antioxidant content should be selectively engineered to reduce oxidative stress. Recent studies have also focused on searching for or creating antioxidant peptides [41,52]. Peptides, most of which are food-derived antioxidant peptides, are thought to promote the health and disease preventing. Although most peptides are extracted from milk or fish, understanding how proteins prevent ROS would also help to generate new proteins with highly oxidative reacting ability.

## Conclusions

The current work proposed a novel SCM-based SCMPSP method for predicting and analysing of PSPs from their sequences. Several other homology-based and machine-learning approaches have been explored: BLAST, support vector machine (SVM), decision tree J48 and Bayes. The performance of the SCMPSP method was comparable to that of the SVM-based method, which in turn outperformed J48- and Bayes-based methods when applied to independent test set. Additionally, the propensity scores derived from the SCMPSP resulted in identification of informative physicochemical properties, providing insights into the nature of PSPs. Our SCM_PCPs method yielded high correlation results with such PCPs, as: BLAS910101, PUNT030101 and WOLR810101. Additional correlation analysis has been conducted to explore the nature of PSPs in their interaction with ROS. In summary, PSPs are more likely to be composed of amino acids with hydrophobic, and electron-reactive side chains, as well as those, which reinforce the formation of helices in membrane environments. Moreover, PSPs have low interaction with water. The SCMPSP source code and the datasets used in this study are available at http://iclab.life.nctu.edu.tw/SCMPSP/.

## Competing interests

The authors declare that they have no competing interests.

## Authors' contributions

TV conceived the idea of this work, carried out the system design, and participated in manuscript preparation. TV and YFL analyzed the physicochemical properties and the protein visulization. PC and HAC implemented the programs and HAC established the website. HLH and SYH participated in the system design, supervised the whole project and coordination, and helped to write the manuscript. All authors have read and approved the final manuscript.

## Declaration statement

This work was funded by National Science Council of Taiwan under the contract numbers of NSC-103-2221-E-009-117- and NSC-103-2221-E-009-141-. The funders had no role in study design, data collection and analysis, decision to publish, or preparation of the manuscript.

This article has been published as part of *BMC Bioinformatics *Volume 16 Supplement 1, 2015: Selected articles from the Thirteenth Asia Pacific Bioinformatics Conference (APBC 2015): Bioinformatics. The full contents of the supplement are available online at http://www.biomedcentral.com/bmcbioinformatics/supplements/16/S1
